# The Role of t(11;14) in Tailoring Treatment Decisions in Multiple Myeloma

**DOI:** 10.3390/cancers15245829

**Published:** 2023-12-13

**Authors:** Martina Kleber, Ioannis Ntanasis-Stathopoulos, Evangelos Terpos

**Affiliations:** 1 Department of Internal Medicine, Clinic Hirslanden Zurich, 8032 Zurich, Switzerland; martina.kleber@hirslanden.ch; 2 Faculty of Medicine, University of Basel, 4031 Basel, Switzerland; 3 Department of Clinical Therapeutics, School of Medicine, National and Kapodistrian University of Athens, 11527 Athens, Greece

**Keywords:** translocation (11;14), prognosis in multiple myeloma, treatment strategies

## Abstract

**Simple Summary:**

The presence of t(11;14) in MM is a significant genetic abnormality and plays an essential role in tailoring treatment decisions. t(11;14) is present in 15% to 20% of MM and is associated with an upregulation of cyclin D1, leading to tumor cell proliferation. Patients harboring t(11;14) often exhibit clinical characteristics that position them between standard- and high-risk categories. Moreover, t(11;14) is associated with higher levels of BCL2, and published data demonstrate that t(11;14) is predictive of the BCL2 dependency. A better understanding of this genetic abnormality influences the choice of therapeutic approaches. In recent studies, the impact of t(11;14) on treatment responses was evident, which emphasize its role in shaping therapeutic strategies. In this review, we highlight the association of t(11;14) with other molecular abnormalities, raise questions about the predictive and prognostic value of t(11;14) in MM and summarize current clinical studies of BCL2 inhibitors in terms of efficacy and safety.

**Abstract:**

Multiple myeloma (MM) represents a hematological neoplasia with an uncontrolled proliferation of malignant plasma cells and complex cytogenetic abnormalities. t(11;14) has emerged as a crucial genetic aberration and is one of the most common primary translocations in MM. Patients harboring t(11;14) represent a distinctive subgroup with a clinical profile that differs from t(11;14)-negative MM risk categories. One of the key features linked with t(11;14) is the BCL2 dependency, indicating vulnerability to BCL2 inhibition. BCL2 inhibitors, such as venetoclax, demonstrated impressive efficacy alone or in combination with other anti-myeloma drugs in patients with RRMM accompanied by t(11;14) and BCL2 overexpression. Therefore, t(11;14) plays a key role in both risk stratification and informed decision making towards a tailored therapy. In this review, we highlight the biology of t(11;14) in MM cells, summarize the current evolving role of t(11;14) in the era of novel agents and novel targeted therapies, illuminate current efficacy and safety data of BCL2-based treatment options and explore the future prospects of individualized precision medicine for this special subgroup of patients with MM.

## 1. Introduction

Multiple myeloma (MM) is a plasma cell dyscrasia characterized by an uncontrolled clonal proliferation of malignant plasma cells and a marked degree of molecular and clinical intricacy [1]. The increased understanding of the disease biology and pathogenesis of MM over the recent years have fundamentally reshaped the methodology in approaching its diagnosis and treatment and have improved patient outcomes [2,3,4,5,6,7].

Translocation and structural and numeric chromosomal irregularities occur not only at the onset of this disease, but also during the course of disease progression or relapse; therefore, they have profound prognostic significance on clinical parameters, as well as a predictive value in terms of the response to different therapeutic regimens during the disease’s course [8,9]. Hence, the revised International Staging System (R-ISS) was developed for risk stratification in newly diagnosed patients with MM based on biochemical markers and genetic abnormalities, such as translocation (t) of chromosome 4 and 14, t(14;16) and the deletion of chromosome 17p (del17p), and has gained a reputation as one of the most important cornerstones in the area of prognostic determinants [10]. The second revised ISS classification (R2-ISS) integrated the 1q gain/amplification in the prognostic risk score classification of patients with newly diagnosed MM (NDMM) [11,12]. The most common 14q32 translocation represents about 60% of all patients with MM and includes t(11;14), t(4;14), t(14;16), t(14;20) and t(6;14) [2,13]. Albeit t(4;14), t(14;16) and t(14,20) are defined as high-risk cytogenetic prognostic markers and are associated with poor prognosis, patients with MM with t(11;14), t(6;14) and/or hyperdiploidy are categorized into the standard-risk disease class [2,13,14]. Thus, studies conducted prior to the novel agent era indicated that patients with t(11;14) are associated with standard-risk profiles. More recent studies have suggested that patients with t(11;14) may exhibit a less favorable response to current treatment regimens compared to standard- or high-risk cytogenetic abnormalities, resulting in diminished survival outcomes [15,16,17].

MM cells carrying t(11;14) have a distinct biological characteristic depicted by increased levels of the anti-apoptotic protein BCL2 and a reduced expression of the pro-apoptotic proteins MCL1 and BCLXL, presenting a unique contrast to MM cells devoid of this specific genetic alteration [18]. Thus, t(11;14) arose as a predictive marker in patients with MM with a vulnerability to BCL2 inhibition [17].

Due to the growing clinical significance of t(11;14) among the numerous genetic abnormalities in MM, we aim to review the biology and the emerging role of t(11;14), its prognostic significance and how it enlightens the current therapeutic strategies.

## 2. Genetic Complexity of Cytogenetic Aberrations and the Role of t(11;14) in MM

### 2.1. Cytogenetic Abnormalities and Their Classification in MM

MM is characterized as a clinically and genetically heterogeneous disease. Cytogenetic aberrations refer to structural or numerical abnormalities and can be divided into hyperdiploidy of odd-numbered chromosomes and translocation including chromosome 14 [17]. The translocation between chromosome 11 and 14 is one of the well-known genetic abnormalities and is present in ~20% of newly diagnosed patients with MM (NDMM), followed by t(4;14) (10% to 20%), t(14;16) (5%), t(14;20) and t(6;14) which lead to the overexpression of CCND1, FGFR3/MMSET, MAF and CCND3 oncogenes, respectively [17,19]. The presence of translocations including chromosome 14, such as t(4,14), t(14;16) and t(14;20), as well as other genetic gains and deletions (del(17p), del(1p32), del(1p12) and amp(1q)), have been associated with dismal patient outcomes, as depicted in the R-ISS and the R2-ISS risk stratification systems [10,11,19]. In contrast, patients with hyperdiploidy and t(6;14) are classified into standard-risk disease, leading to an extended remission rate and prolonged survival outcome. While t(11;14) was formerly categorized as a standard-risk cytogenetic aberration, the latest studies with novel agents suggest that newly diagnosed patients with MM carrying t(11;14) have an unfavorable prognosis than was indicated in earlier findings (Table 1) [15,20,21,22].

### 2.2. t(11;14) in MM: Biology and Clinical Findings

The translocation t(11;14) engages IGH in chromosome 14 and has been associated with increased cyclin D1 (CCND1) expression. Moreover, one-third of these patients harbor additional high-risk abnormalities (e.g., del13q in 37%, del(IGH) in 33%, gain1q in 20%, del16q in 15%) [27]. In addition, t(11;14) confers unique cellular features, such as lymphoplasmacytic phenotype and an increased numbers of circulating plasma cells, and often lacks traditional plasma cell markers compared to other MM cell types. The expression of the B-cell lineage membrane protein CD20, increased levels of the B-cell receptor CD79a and decreased expression of CD38 and CD56 have been detected in patients with MM with t(11;14) [28]. MM cell lines with t(11;14) have a high expression of the anti-apoptotic protein BCL2, which is a crucial factor to consider when making treatment decisions. Patients with MM sheltering t(11;14) present a unique clinical characteristic and cell biology. Apart from lymphoplasmocytic morphology, the expression of cyclin D1 and CD20 is perhaps the preclinical feature of the greatest significance combined with an elevated expression of the anti-apoptotic protein BCL2; this provides a potential opportunity for targeted therapies.

Numerous common clinical features in patients with t(11;14) have been described since patients with MM with t(11;14) have a unique myeloma type and a lower probability to be hyperdiploid [20]. Interestingly, a higher incidence of t(11;14) in younger patients less than 50 years has been reported, whereas t(11;14) has already been reported in patients with monoclonal gammopathy of an undetermined significance, indicating that this could be an early event in the pathogenesis of the disease [29,30]. In addition to the above, patients diagnosed with plasma cell leukemia or light-chain AL amyloidosis have a high incidence of t(11;14) reaching 40% or 50% of the cases, respectively [31]. Furthermore, patients with t(11;14) have a higher rate of myeloma bone disease, light-chain-only myeloma, non-secretory disease and renal impairment due to cast nephropathy compared to patients without t(11;14), whereas t(11;14) is a typical characteristic of patients with IgM myeloma [16,32].

### 2.3. Dynamic Role of t(11;14) in Shaping the MM Prognosis Landscape in the Era of Novel Agents

In the pre-novel era, initial studies assessing the outcomes and prognosis of patients with MM with t(11;14) exhibited relatively small patient samples and were subject to certain constraints. The assessment of translocation used conventional cytogenetics and combined the analysis of 11q abnormalities [33,34]. In the era of fluorescence, in situ hybridization (FISH) analysis of larger published studies suggested that patients with t(11;14) have equivalent outcomes compared to patients without t(11;14) [21,22]. In line with this, the Mayo Stratification of Myeloma and Risk-Adapted Therapy consensus guidelines (mSMART), which is predominantly based on cytogenetic features to predict risk stratification, classified t(11;14) as a standard-risk cytogenetic abnormality [35,36].

The prognosis of patients with newly diagnosed (NDMM) and relapsed/refractory (RRMM) MM in terms of overall survival (OS) has improved with the advent of targeted drugs, such as proteasome inhibitors (PIs) and immunomodulatory agents (IMiDs) [37,38,39]. However, outcomes in patients with t(11;14) vary, often irrespectively of their standard stratification according to standard- or high-risk cytogenetic abnormalities. A large study of patients with t(11;14) MM with no high-risk cytogenetics demonstrated significantly shorter progression-free survival (PFS) results in comparison to patients without t(11;14) and no high-risk cytogenetics [25]. Moreover, the effect of t(11;14) on the outcome was evaluated in patients treated with novel agents during the induction and maintenance phase of treatment (Table 1) [15,23,40]. A study by Sasaki et al. compared t(11;14) with high-risk cytogenetic abnormalities and standard-risk cytogenetics in patients with MM undergoing autologous stem cell transplantation (ASCT) [15]. After a follow-up of 37 months, the 3-year PFS in patients with t(11;14) vs. standard cytogenetics and high-risk cytogenetics was 27% vs. 47% vs. 13%, respectively (*p* ≤ 0.00001). In addition, the 3-year OS was highest in patients with MM with standard cytogenetics, followed by patients with t(11;14) and being the lowest in patients with high-risk cytogenetics (83%, 63% vs. 34%, respectively, *p* ≤ 0.00001). Importantly, in these initial studies, translocation and high-risk cytogenetics were analyzed by conventional cytogenetics or FISH, and 66% to 78% of these patients were treated with induction therapy based on novel agents. One of the first large studies analyzing the outcome of 1000 patients with NDMM with t(11;14) and homogeneously treated with bortezomib, lenalidomide and dexamethasone (VRd) followed by ASCT was initiated by the Emory group [23]. The results revealed that the response rates (very good partial response or better) after induction were lower in patients with t(11;14) compared to patients with standard-risk disease (50% vs. 76%, respectively, *p* < 0.001). Furthermore, these response rates were translated into inferior median PFS in patients with t(11;14) of 51 months vs. 75 months for those who did not harbor t(11;14) (*p* < 0.001), respectively.

Likewise, a study from the Mayo Clinic compared the outcomes of 365 patients with t(11;14), 132 patients with MM with non-t(11;14) and 598 patients with no translocation [16]. Partial response rates (or better) were the lowest in patients with t(11;14) compared to non-t(11;14) or no translocation with 72% vs. 82% and 85%, respectively. Similarly, median PFS was 23 months in the t(11;14) group compared to 19 months vs. 28 months in non-t(11;14) vs. no translocation, respectively. Moreover, the OS rate for t(11;14) was higher compared to patients with the non-t(11;14) translocation, but was inferior to those without any translocation (74 months vs. 50 months vs. 104 months, respectively; *p* < 0.01). These differences also remained the same after excluding patients with MM with del(17p) from all subgroups. The results indicated that t(11;14) behaved rather like an “intermediate-risk” disease, since all outcomes were inferior compared to the patients with no translocation and superior to those with non-t(11;14). The impaired effectiveness of PIs in inducing endoplasmic reticulum stress [24] and the negative impact of bortezomib-based therapy regimens in t(11;14) light-chain amyloidosis could be responsible for the inferior response rates in these special patient cohorts [41,42].

Since patients with t(11;14) were almost analyzed in patients with hyperdiploidy and MM, which are categorized as suffering from a standard-risk disease, the impact of ASCT in these patients is not sufficiently clarified. Due to the results of the Mayo Clinic study, which showed an increased OS in patients with early ASCT compared to those with a delayed ASCT (irrespective of cytogenetic abnormalities [16] and the impaired response in these patients treated with PIs or IMIDs-based combinations), an early ASCT in transplant-eligible patients with t(11;14) could be preferred. In contrast, former studies within the Connect registry including first-line therapy with PIs and IMiDs alone or combined in NDMM revealed no differences in the outcome with or without t(11;14) (Table 1) [26].

Taking all the above into consideration, we should re-define the risk classification of t(11;14). Patients with this translocation seem to have inferior outcomes in terms of ORR and survival compared with standard-risk patients with MM [16,23,40]. Importantly, it has been shown that the presence of numerous high-risk cytogenetic abnormalities in the same patient, such as multiple gains(1q)m del(1p)m del(IGH) and del(13q), has a greater impact on outcomes compared to a single high-risk aberration such as an isolated t(11;14) [43,44]. Therefore, we advocate for patients with t(11;14) and co-existing chromosomal abnormalities to be categorized as at least intermediate-risk than standard-risk [16,45].

## 3. Involvement of the BCL2 Family in MM

BCL2 belongs to the regulatory family that governs apoptosis by initiating or halting the process of cell death [46]. The equilibrium in programmed cell death is maintained through the fragile balance of anti-apoptotic proteins such BCL2, BCLXL, BCLW and MCL1 and pro-apoptotic BAX, BAK, BIK (BH3 domain containing proteins), BIM and BAD proteins [17,47]. The BCL2 family is either pro-apoptotic (such as BIM, PUMA, NOXA) or anti-apoptotic (e.g., BCL2, BCLXL, MCL1). The decision if cells are undergoing cell death depends on the balance of these proteins [17].

A specific characteristic of all BCL2 family proteins is the existence of BH-binding domains, mostly BH3, which plays a central role for cell death within the BCL2 family [48]. Initial preclinical data revealed a relevant significance of BH3 mimetic agents in myeloma cells lines with t(11;14), which has a high BCL2/MCL1 ratio [49]. Moreover, BH3 profiling assays were linked to MM cell lines on BCL2 for survival, which was pronounced in myeloma cells harboring t(11;14) abnormalities [50]. While nearly 80% of myeloma cells lines are dependent on the anti-apoptotic MCL1 protein or both MCL1 and BCLXL, which is an important survival protein in MM [46], MM with t(11;14) biology is characterized by an increased expression of BCL2 expression and diminished MCL1/BCLXL expression [46,51]. Conversely, only 20% of myelomas preferentially transmit signals via BCL2 proteins, which could be targeted by venetoclax [46]. The responses to BCL2 inhibitors are based on anti-apoptotic properties for survival; thus, the upregulation of BCL2 serves as a distinctive trait of resistance to apoptosis.

The impact of venetoclax is predominantly defined by an inhibition of BCL2, not BCLXL or MCL1, and has provided single agents effects, suggesting BCL2 dependence in some MM cells [18]. Moreover, due to the substantial response rates of venetoclax in patients with relapsed/refractory myeloma (RRMM) with t(11;14), the BCL2 dependency seems to be a major driver for the progression of the MM disease, also within the clonal evolution.

Patients with relapsed MM showed an increased expression of BCLXL; therefore, in cases of resistance to BCL2 inhibition with venetoclax, a combination of BCL2 and BCLXL inhibitors presents an effective alternative, indicating that BCLXL dependence is also a pro-survival factor in MM [51].

The expression of MCL1 is associated with MM relapse and a diminished survival. Underlined by the fact that an certain amount of MM cell lines are more sensitive to MCL1 inhibition and rather less sensitive to BCL2 and BCLXL inhibition, MCL1 might be a superior target than BCL2 [52].

## 4. Therapeutic Implications and Tailoring a Treatment Decision in Patients with MM with t(11;14)

Deeper insights into the biology of plasma cells with t(11;14) and its prognostic implications enable the development of ongoing and future therapeutic strategies. Thus, for tailoring a treatment decision towards a personalized therapy strategy, the assessment of the status of t(11;14) in patients with MM is essential.

### 4.1. BCL2 Inhibitors

#### 4.1.1. Venetoclax

The first results of a phase I clinical trial demonstrated significant clinical activity of the t(11;14)-targeted agent venetoclax, an oral BCL-2 inhibitor, in heavily pretreated patients with RRMM [53] (Table 2). Patients had a median of five prior lines of therapy, including 61% of refractory patients to both PIs and IMiDs. Of note, 46% were t(11,14) positive and achieved an overall response rate (ORR) and ≥vgPR rate of 40% and 27%, respectively. Moreover, gene expression analysis results showed that the ratio of BCL2:BCLXL and BCL2:MCL1 were markedly increased in patients who responded to venetoclax. Due to these results with a high frequency of BCL2 overexpression, the role of t(11;14) as a predictive marker could be established.

Another phase 1b study administered venetoclax in combination with bortezomib and dexamethasone in 66 patients with MM, of whom 39% were refractory to bortezomib and 53% refractory to lenalidomide [59]. The ORR was 75% and 65% in patients with and without t(11;14), respectively. Interestingly, the ORR was nearly 30% among 26 patients who were refractory to bortezomib. Among those who responded to treatment, 38% were positive for t(11;14), suggesting that they may have responded primarily to venetoclax.

The results underline that besides the single agent activity, venetoclax may enrich the level of apoptosis triggered by additional anti-MM cells. Since PIs upregulate the BH3-only protein NOXA (anti-apoptotic protein binding inhibiting the MCL1 activity), the combination of PI with venetoclax and dexamethasone may lower the resistance to venetoclax activity by decreasing MCL1 activity [62].

Furthermore, in the phase III BELLINI trial, venetoclax (800 mg/d) was coupled with bortezomib and dexamethasone [60]. In total, 291 individuals with MM were randomly assigned to receive venetoclax, bortezomib and dexamethasone or a placebo with bortezomib and dexamethasone in this trial. Patients in the venetoclax group had a PFS of 37 months after a median follow-up of 4 years, compared to 9.3 months in the placebo group (HR: 0.12; 95% CI, 0.03–0.44; *p* = 0.0014). While patients with t(11;14) or a higher BCL2 expression showed the greatest benefits, the median OS in both subgroups was not attained (HR, 0.61; 95% CI, 0.16–2.32; *p* = 0.4654) [60,61]. The BELLINI trial was stopped because of the higher death rates from infectious complications in the venetoclax arm over the course of the disease (40%, 78 fatalities), which were limited to patients with MM with a reduced BCL2 expression in the non-t(11;14) group. Given the higher prevalence of OS events in individuals treated with venetoclax in the BELLINI study in 2019, the US Food and Drug Administration placed a partial clinical hold on trials including the medication in patients with RRMM. Only patients with RRMM with t(11;14) were given venetoclax in a biomarker-driven approach after the hold was loosened.

Additionally, in patients with RRMM with and without t(11;14), a phase II non-randomized trial assessed the effectiveness of venetoclax in combination with carfilzomib and dexamethasone [55]. In total, 49 individuals were included with a median of one (range 1–3) previous treatment line. The overall cohort showed an ORR of 80%, and in patients with t(11;14), it rose to 92% from 75% in the non-t(11;14) group. A median PFS of 23 months was reported, whereas 41% of the patients achieved at least complete remission (CR). The primary results of the randomized part of this study were presented in the recent International Myeloma Workshop [63]. Patients with RRMM who were positive for t(11;14) were randomized in a ratio 5:3:5 to receive carfilzomib at 70 mg/m^2^ weekly with dexamethasone at 40 mg and venetoclax at either 400 mg or 800 mg daily, or carfilzomib with dexamethasone (Kd) without venetoclax. The ORR was 94%, 95% and 58% for venetoclax 400 mg with Kd and venetoclax 800 mg with Kd and Kd without venetoclax, respectively. The median PFS was 42.4 months in the group receiving venetoclax 800 mg with Kd and it was not reached in the other groups. Common treatment-emergent toxicities included diarrhea (65% versus 75% versus 6% for Ven400Kd versus Ven800Kd versus Kd groups, respectively), nausea (53% versus 55% versus 28%, respectively), fatigue (35% versus 50% versus 22%, respectively) and vomiting (0 versus 50% versus 11%, respectively). Furthermore, severe grade ≥3 infection rates were higher in the VenKd groups compared to the Kd combination (29% versus 20% versus 11%, respectively) [63].

Anti-CD38 monoclonal antibodies were introduced into the whole disease course of myeloma from newly diagnosed disease to relapsed/refractory myeloma [64]. Therefore, the combination of venetoclax with anti-CD38 treatment may further enhance patient outcomes. Bahlis et al. investigated the efficacy of venetoclax with daratumumab and dexamethasone (VenDd, part 1) in a three-part phase I/II study in patients with RRMM with t(11;14) and venetoclax, daratumumab, bortezomib and dexamethasone (VenDVd, part 2), irrespective of cytogenetics abnormalities [65]. Among the 48 enrolled patients, the primary objective was safety and ORR. The results with a median follow-up of 21 months in the VenDd vs. 20 months in the VenDVd group revealed ORR rates of 96% in the VenDd and 92% within the VenDVd group, respectively. This study showed no new safety issues and confirmed that venetolax is more effective in patients with t(11;14), although the efficacy results were encouraging in the unselected patient population as well. Updated results of the part 3 of this study (NCT03314181) showed an acceptable safety profile and deeper response rates of VenDd versus DVd in patients with t(11;14) and RRMM who had received at least 1 prior line of therapy, including an IMiD, and were non-refractory to PIs or anti-CD38 antibodies [54]. The ORRs were 95%, 100% and 62% for the Ven400Dd, Ven800Dd and DVd arms, respectively. The rates of VGPR or better were 86%, 100% and 38%, respectively. Similarly, the 24-month PFS rate was 94% (95% CI 63.2, 99.1), 83% (95% CI 27.3, 97.5) and 47% (95% CI 20.6, 70.2) for the Ven400Dd, Ven800Dd and DVd arms, respectively.

Based on the results of the BELLINI study, currently, the investigations of venetoclax are constrained to t(11;14)-positive patients. The phase 3 multicenter, randomized CANOVA trial (NTC0353944) compared the efficacy and safety of venetoclax with dexamethasone compared with the combination of pomalidomide with dexamethasone in t(11;14)-positive patients with RRMM with the primary endpoint of PFS [57]. The primary results were presented in the recent IMS meeting and showed an ORR of 62% in the venetoclax group compared with 35% in the pomalidomide group (*p*-value < 0.001). However, the primary endpoint of PFS superiority was not met (9.9 versus 5.8 months, for the venetoclax and pomalidomide groups, respectively, *p*-value 0.24). The median OS was 32.4 months in the venetoclax group and 24.5 months in the pomalidomide group (*p*-value 0.07) [58].

The currently published data underline the significance of BCL2-directed therapy, such as venetoclax in patients with t(11;14), constituting the initial step towards a targeted treatment strategy in MM with t(11;14). It should be taken into consideration that the investigations with venetoclax are still ongoing, and the current results should be observed in the context of clinical trials. Still, many unanswered questions regarding the use of venetoclax remain, such as the optimal duration of therapy, its use as a first-line treatment and how to handle resistance to venetoclax. Venetoclax is not universally effective in all patients with t(11;14), and resistance could occur de novo or during the course of the disease; moreover, an increased BCL2 expression correlates with an increased response to venetoclax [66]. Nevertheless, previous data showed that MCL1 and BCLXL expressions are associated with a diminished response [51]; current studies with BH3 suggest that MM varied in terms of its dependency on anti-apoptotic proteins [51]. In contrast to the currently published data, it could be demonstrated that t(11,14) and CCND1 may not directly have an impact on the response to venetoclax, and additional predictive factors for this response were identified, such as an increased expression of B-cell genes [67].

Of note, a special cohort of patients without t(11;14) could also respond to venetoclax. This could be observed in high-risk patients with a genetic abnormality of t(14;16) [67]. The results revealed that in the absence of t(11;14), t(14;16) positivity was associated with an exceptional response to venetoclax due to an increased CD2 expression [67].

Moreover, published data suggest that plasma cell phenotypes in patients with MM with t(11;14) had significantly increased levels of CD79A and PAX5, decreased levels of CD38 and CD138 expression and an elevated BCL2/BCL2L1 ratio, indicating vulnerability to venetoclax and fewer effects of daratumumab [68]. Interestingly, venetoclax resistance in patients with non-t(11;14) and MM is connected with a high expression of neuregulin-2 [69]. Moreover, a de novo D111A mutation in BCL2 was identified in patients with MM progressing on venetoclax. Preclinical studies have shown that this mutation confers resistance to the anti-myeloma activity of venetoclax [70]. Furthermore, a transition of myeloma cells from BCL2 to MCL1/BCLXL dependence may lead to a resistance to venetoclax as well [71].

#### 4.1.2. AT-101

AT-101 is an oral inhibitor of BCL2 and MCL1 with lesser effects on BCLXL and BCLW [72]. Preclinical data on MM suggest that AT-101 has synergistic cytotoxic effects with lenalidomide and dexamethasone by disturbing the function of BCL2 and MCL1 [73]. The first results were provided starting from a phase I study analyzing the efficacy of AT-101, lenalidomide and dexamethasone in patients with RRMM with a median of two prior lines of therapy. Albeit t(11;14) was only evident in 1/10 patients and 30% of the patients were lenalidomide-refractory, 20% bortezomib-refractory and 30% daratumumab-refractory, and the ORR was 44% with a median PFS of 8 months, underlining the necessity of additional investigations of AT-101 therapy in patients with RRMM.

#### 4.1.3. APG-2575, BGB-11417 and AZD-0466

APG-2575 (Lisaftoclax) is a selective and potent BCL2 inhibitor that competes with BIM in the BCL2/BIM complex. Although this investigation focuses on other hematological malignancies [74], several trials in RRMM are underway (NCT04942067, NCT04674514).

Patients with non-Hodgkin lymphomas (NHL) and CLL/SLL have already demonstrated efficacious antitumor activity with BGB-11417, a selective BCL2 inhibitor [75]. BGB-11417 alone or in combinations is being investigated in patients with RRMM. In patients with t(11;14) and RRMM, a phase Ib/II trial (NCT04973605) examines the safety and effectiveness of BGB-11417 in combination with dexamethasone and/or carfilzomib in combination with dexamethasone. PI, IMiD and anti-CD38 therapy-exposed eligible individuals with t(11;14)-positive RRMM were included. Patients were given 80 mg, 160 mg, 320 mg or 640 mg of BGB-11417 per day along with 40 mg of dexamethasone per week. No dose-limiting toxicity occurred. Up until now, high-dose cohorts of BGB-11417 with dexamethasone totaling 80 mg, 160 mg, 320 mg (3 patients each) and 640 mg (1 patient) have been administered to 10 patients. Enrolled patients had a median age of 69 years (range 52–81 years) and a median of 3 (range 1–5) prior lines of therapy. The median duration of treatment was rather short (3.2 months, range 0.5–6.5). Three patients died. A grade 3 elevation in liver enzymes and lymphopenia was reported in two patients. Other frequent adverse events included insomnia (50%), exhaustion (30%) and arthralgia (20%) [76].

ABT-737 demonstrated affinity for the BH3 binding site of BCL2, BCLXL and BCLW in preclinical and clinical investigations of solid tumors [77]. The orally bio-available navitoclax (ABT-263) was halted due to significant BCLXL-dependent apoptosis of platelets, resulting in significant thrombocytopenia [78]. The additional BCLXL affinity combined with BCL2 is of potential special interest in patients with MM with venetoclax resistance, since increased levels of BCLXL were found in myeloma xenograft model with venetoclax resistance [51]. Since AZD-4320 (a combined BCL2/BCLXL inhibitor) showed transient thrombocytopenia but increased cardiac toxicity, a novel conjugated AZD-4320 was developed with in vitro and in vivo efficacy in hematological malignancies [79]. Currently, a phase I/II study analyzing the efficacy and safety of AZD-0466 alone or in combination in relapsed hematological malignancies including RRMM is underway (NCT04865419).

### 4.2. MCL1 Inhibitors

MLC1 expression is essential for the survival of plasma cells, and increased MCL1 levels in plasma cells correlate with an increased risk for relapse and diminished OS [80]. The upregulation of MCL1 is also involved in the mechanism of venetoclax resistance [81], highlighting MCL1 as a potential target in the treatment of patients with RRMM. Currently, several studies are ongoing with MCL1- or combined MCL1 and BCL2 inhibition. Early clinical trials are currently investigating MCL1 inhibitors such S64315, AZD5991, AMG176 and AMG397 [82,83,84].

## 5. Conclusions and Future Directions

MM is a genetically complex and heterogeneous plasma cell dyscrasia. The increased insights into the heterogeneity with genetic abnormalities, clinical presentation and treatment response have led to a paradigm transition in the understanding of the MM disease.

The t(11;14) translocation, which occurs in 15% to 20% of patients with MM and is currently categorized as a standard-risk abnormality, became a significant center of attention in the landscape of this malignancy. Due to the results with inferior outcomes in patients with t(11;14) treated with novel agents, its classic categorization has faced opposition. The distinct molecular and clinical features of t(11;14), such as the overexpression of BCL2 and the high ratio of BCL2/MCL1, indicate a vulnerability to BCL1 inhibitors.

While various treatment options are currently available, there is an ongoing clinical need for predictive biomarkers guiding therapy decisions. With the introduction of BCL2 inhibitors, t(11;14) emerged as a primary predictive biomarker in patients with MM, poised to reshape the therapeutic landscape for this specific subgroup of patients. Although we traditionally classify risk groups in MM as standard- or high-risk, patients with t(11;14) exhibit outcomes that fall between these two extremes. Moreover, multiple studies on patients with t(11;14) and MM consistently demonstrated inferior outcomes compared to hyperdiploid standard-risk patients. In addition, t(11;14) showed a strong association with additional molecular and chromosomal abnormalities.

Understanding the role of t(11;14) can also guide the development of targeted therapies in MM and may thus improve the outcomes of this subgroup of patient with MM. Therefore, the presence of this translocation highlights the need for precise clinical decision making. Physicians should consider t(11;14), besides other important genetic markers, when devising treatment strategies for patients with MM. Novel therapies targeting the BCL2 family, such as venetoclax, offer hope for increased responses to therapy and improved survival. Venetoclax has shown ORR responses of about 40% in patients with RRMM harboring t(11;14) and demonstrated synergistic effects if combined with PIs and dexamethasone, which were most improved in patients with MM with t(11;14). The exact definition of patients with MM who probably gain from venetoclax therapy is still ongoing, and determining the characteristics associated with the response to venetoclax is vital.

Several considerations should be taken into account and need to be addressed in future clinical studies or during the development of targeted therapies in this field. First, a global consensus for routinely performing FISH testing of t(11;14) at the time of diagnosis and during relapse of the disease is warranted since t(11;14) demonstrates a rather special biology in comparison to a specific risk group. Second, the transition in the dependence of MM cells from BCL2 to MCL1, as observed in the context of venetoclax resistance, highlights the complex interplay between these proteins. Thus, research should address the mechanisms of the acquired resistance, which will hold the key to developing strategies to overcome treatment hurdles and improve long-term responses. Third, future clinical trials should additionally focus on the incorporation of BCL2 inhibitors into earlier treatment lines, and potent combinations of BCL2 inhibitors in conjunction with other anti-myeloma agents and novel intervention strategies hold the potential to enhance the outcome in both patients with NDMM and patients with RRMM.

The encouraging results of preclinical and clinical data underline the need for BCL2 family gene expression profiling (i.e., use of BCL2 inhibitors) and the recognition of t(11;14) as a predictive biomarker for the responses to therapy. t(11;14) is a key determinant in the individualized approach in MM therapy and enlarges the spectrum of possibilities in precision medicine, advancing tailored treatment options based on patients’ specific molecular and genetic profiles. In the forthcoming years, clinical trials in precision medicine will play a crucial role in enhancing the efficacy of treatments by minimizing potential side effects. Recognizing its significance paves the road for precise therapeutic decisions to improve the current outcome of patients with MM. Thus, in the future, practitioners should focus more on increasing the number of tailored therapies, prognostic accuracies and increasing the understanding of the resistance mechanism to enhance the management of the disease and the quality of life for patients with the myeloma disease.

## Figures and Tables

**Table 1:** Outcomes of patients with NDMM with t(11;14) treated with novel agents.

Reference	Study Details	Regimens	Cytogenetics	Outcomes
Saskia et al. [15] 2013	Retrospective, n = 993	ASCT Induction with 66–78% novel agents	t(11;14): 27 pts HR: 97 pts Standard risk: 869 pts	t(11;14) vs. HR vs. standard risk Three-year PFS: 27% vs. 13% vs. 47%
Lashman et al. [16] 2018	Retrospective, n = 1095	ASCT in 58–61% with a novel agent-based induction of 90%	t(11;14): 365 pts Non-t(11;14): 132 pts No translocation: 598 pts	t(11;14) vs. non-t(11;14) vs. standard PFS 23 vs. 19 vs. 28 months OS 74 vs. 50 vs. 104 months ORR 71% vs. 82% vs. 85%
Joseph et al. [23,24] 2020	Retrospective, n = 1000 NDMM	Induction with RVD followed by ASCT	t(11;14): 14%	t(11;14) vs. standard-risk non-t(11;14) 50% vs. 76% PFS 51 vs. 75 months (*p* < 0.001)
Bal et al. [25] 2021	Retrospective, n = 5581	IMiDs plus PI IMiDs only PI only ASCT	t(11;14) with no HR abnormalities: 589 pts Non-t(11;14) with no HR abnormalities: 2909 pts	t(11;14) vs. non-t(11;14) OS 72 vs. 77 months (*p* = 0.19) PFS 36 months vs. 40 months (*p* = 0.028)
Gasparetto et al. [26] 2022	Prospective observational cohort study, n = 1574	Induction therapy with 80% novel agents followed by ASCT	t(11;14): 378 pts Non-t(11;14): 1196 pts	t(11;14) vs. non-t(11;14) OS 74 months vs. 77 months (*p* = 0.942) PFS 35 months vs. 36 months (*p* = 0.768)

Abbreviation: ASCT: autologous stem cell transplantation; HR: high risk; IMiDs: immunomodulatory agents; NDMM: newly diagnosed multiple myeloma; PI: proteasome inhibitors; pts: patients; RVD: lenalidomide, bortezomib, dexamethasone.

**Table 2:** Clinical trials including venetoclax-based therapy in MM.

Reference	Phase	Regimens	Study Cohort	Median Prior Therapy Lines/Refractoriness	Cytogenetics	ORR	Common Grade 3–4 Toxicity
Kumar et al. [53] 2017	Phase I, non-randomized	Venetoclax (300, 600, 900, 1200 mg/d), MTD nr.	66 pts RRMM	5 lines Len: 77% Borte: 70% Pom: 53% ASCT: 76%	t(11;14): 46% High risk: 27%	21%, 40% in t(11;14)	Thrombocytopenia: 26% Neutropenia: 21% Anemia: 14% Pneumonia: 8%
Kaufman et al. [54] 2020	Phase I/II, non-randomized	Venetoclax 800 mg/d Dexa 40 mg/wk	RRMM 20 pts phase I, 31 pts phase II	3 lines phase I 5 lines phase II	t(11;14)	60% phase I 48% phase II	Phase I: Thrombocytopenia: 10% Neutropenia: 10% Anemia: 10%
Costa et al. [55] 2021	Phase II non-randomized	Venetoclax MTD nr Carfilzomib 70 mg/m^2^ Dexa	RRMM 49 pts	1–3 lines	t(11;14)	92%	No novel safety concerns
Gasparetto et al. [56] 2021	Phase II, non-randomized	Venetoclax 400 mg/d Pomalidomide 4 mg/d Dexa 40 mg/wk	RRMM 8 pts	Len: 75% Borte: not reported	t(11;14): 38% High risk: 63%	63%, 67% in t(11;14)	Neutropenia: 75% Leukopenia: 12%
Mateos et al. [57,58] 2020, 2023	Phase II, randomized, CANOVA trial	Venetoclax400 mg/d Dexa: 40 mg/wk vs. Pomalidomide Dexa 40 mg/wk	RRMM 254 pts	≥2 lines Len-refractory 100%	t(11;14): 100% High risk: 8%	62% VenDex vs. 35% PomDex (*p* < 0.001)	VenDex: any grade infection (61%), diarrhea (41%), lymphopenia (24%), nausea (22%)
Moreau et al. [59] 2017	Phase Ib, non-randomized	Venetoclax: 100–1200 mg/d Borte: 1.3 mg/m^2^ Dexa: 20 mg	RRMM 66 pts	3 lines Borte: 39% Len: 53% ASCT: 59%	t(11;14): 14%	67%	Thrombocytopenia: 29% Neutropenia: 14% Anemia: 15% Pneumonia: 8%
Kumar et al. [60,61] 2020, 2021	Phase III, randomized BELLINI trial	Venetoclax 800 mg/d Borte: 1.3 mg/m^2^ Dexa: 20 mg vs. Borte: 1.3 mg/m^2^ Dexa: 20 mg	RRMM Venetoclax: 194 pts Placebo: 97 pts	1–3 lines	t(11;14): 15%	Venetoclax: 82% Placebo: 68%	Suspended due to safety Death: Venetoclax: 35% Placebo 29% Fatal infection: Venetoclax: 9 pts Placebo: 0

Abbreviation: ASCT: autologous stem cell transplantation, borte: bortezomib, d: day, Dexa: dexamethasone, len: lenalidomide; MDT: maximum tolerated dose, nr: not reached, Pom: pomalidomide, pts: patients; wk: week.

## Data Availability

The data presented in this study are available in this article.
